# Transgenerational hormetic effects of sublethal dose of flupyradifurone on the green peach aphid, *Myzus persicae* (Sulzer) (Hemiptera: Aphididae)

**DOI:** 10.1371/journal.pone.0208058

**Published:** 2019-01-24

**Authors:** Qiuling Tang, Kangsheng Ma, Hsin Chi, Youming Hou, Xiwu Gao

**Affiliations:** 1 State Key Laboratory of Ecological Pest Control for Fujian and Taiwan Crops, Fujian Agriculture and Forestry University, Fuzhou, Fujian, PR China; 2 Department of Entomology, China Agricultural University, Beijing, PR China; 3 Department of Plant Production and Technologies, Niğde Ömer Halisdemir University, Niğde, Turkey; 4 Fujian Province Key Laboratory of Insect Ecology, Department of Plant Protection, Fujian Agriculture and Forestry University, Fuzhou, Fujian, PR China; Institut Sophia Agrobiotech, FRANCE

## Abstract

Both inhibitory and stimulatory (known as hormesis) effects of the sublethal flupyradifurone, a butenolide insecticide, on *Myzus persicae* Sulzer (Hemiptera: Aphididae) were investigated for incorporating it into integrated pest management (IPM). A leaf-dip bioassay showed that flupyradifurone was very toxic against adult *M*. *persicae* with a 48 h LC_50_ of 8.491 mg/L. Using the age-stage two-sex life table approach, we assessed the effects of LC_25_ of flupyradifurone on adult *M*. *persicae* and its progeny (F_1_ and F_2_). On the one hand, aphids exposed to flupyradifurone had significantly negative effects on the life history traits acrossing the generations, such as reduced the adult longevity and fecundity of F_0_, shortened the duration of third instar and fourth instar nymphs, preadult period and the pre-reproductive period of F_1_, and decreased the reproductive days and adult longevity of F_2_. On the other hand, stimulatory effects on the duration of pre-adult, adult reproductive days, and reproduction of F_1_ were observed in the flupyradifurone-treated aphids. Consistently with the stimulation on individual traits, a higher net reproductive rate (*R*_0_) of F_1_ and a shorter mean generation time (*T*) of F_2_ were observed in the flupyradifurone-treated aphids, although the other population parameters including the intrinsic rate of increase (*r*), finite rate of increase (*λ*) and *T* of F_1_ and *R*_0_, *r* and *λ* of F_2_ were not significantly affected. These results revealed that adult *M*. *persicae* exposed to sublethal concentration of flupyradifurone can induce hormetic effects on F_1,_ and also cause negative effects on F_2_. Our results would be useful for assessing the overall effects of flupyradifurone on *M*. *persicae* and the hormetic effects should take into consideration when use flupyradifurone for control *M*. *persicae*.

## Introduction

The green peach aphid, *Myzus persicae* Sulzer (Hemiptera: Aphididae), is one of the most destructive and cosmopolitan insect pest of economical crops [1]. The green peach aphid feeding can cause direct damage and may cause indirect damage through the transmission over 100 plant pathogenic viruses [2]. Control of *M*. *persicae* has been dependent the use of chemical insecticides which frequently resulted in development of resistance to various classes of insecticides, including organophosphates, carbamates, pyrethroids, and neonicotinoids [3,4]. Therefore, the insecticides with environment safety and different modes of action remain critical for control of *M*. *persicae*.

The novel butenolide insecticide flupyradifurone was discovered and developed by Bayer CropScience in 2012 [5]. Flupyradifurone acts as a partial agonist on insect nicotinic acetylcholine receptors (nAChRs) and reversibly binds to acetylcholine (ACh) [6,7], but is structurally distinct from the class of neonicotinoid insecticides [7]. It was introduced as an effective insecticide to control a broad range of sucking pests and lacks significant cross resistance to both imidacloprid and pymetrozine in *CYP6CM1-*mediated resistance of whiteflies [7,8]. Especially, it shows an excellent safety profile for honey bees [5]. Insects are exposed to both lethal and low or sublethal concentrations of insecticide residues under field conditions due to misapplication, pesticide drift, shielding by vegetation, or residual levels after dissipation in the environment [9], thus they may experience directly mortality and certain related sublethal effects [10,11]. Sublethal effects are defined as physiological and/or behavioral effects on individuals that survived from exposure to a pesticide at sublethal concentration [10,12]. On the one hand, sublethal effects of insecticides could affect population dynamics through impaired behaviors and physiological traits, such as reduce insect longevity and fecundity [10,13]. On the other hand, sublethal effects could increase fecundity after exposure to an insecticide and have been documented in several insect pests, such as *M*. *persicae* [14–17], *Rhopalosiphum padi* L. (Hemiptera: Aphididae) [18], and *Frankliniella occidentalis* Pergande (Thysanoptera: Thripidae) [19]. This stimulatory effects induced by insecticides is called “insecticide-induced hormesis”. Hormesis is a biphasic dose–response phenomenon characterized by a low-dose stimulation and a high-dose inhibition and this hormetic response is a modest overcompensation to a disruption in homeostasis or of a direct stimulatory nature [20–24]. It was an adaptive response of generally similar quantitative features with respect to amplitude and range of stimulatory response [21,25–27]. For example, the sublethal doses of imidacloprid and precocene can induce stimulation of reproduction of *M*. *persicae*, however, inhibition at high doses [14,16]. Flonicamid and thiamethoxan can prolong the duration of phloem ingestion of *M*. *persicae* at sublethal doses, whereas induce starvation or contact toxicity at high doses [28]. Further, some survivors with hormesis may develop resistance to the insecticide subsequently [18,19,29,30] and induce pest resurgence and outbreak [24,31,32].

Therefore, it is important to understand the sublethal effects on non-targeted arthropods and overall effects on targeted pests caused by pesticides. The flupyradifurone was recently introduced in markets and has proved to be especially effective against a wide range of homopterous insect pests, including *M*. *persicae* [7]. However, to date, the potential sublethal or hormetic effects of flupyradifurone on *M*. *persicae* are still unknown. To obtain a comprehensive understanding of the overall effects of flupyradifurone on *M*. *persicae*, we assessed the effects of a sublethal concentration (LC_25_) of flupyradifurone on biological traits and demographic parameters of *M*. *persicae* using the age-stage, two-sex life table. This information would be important to enable a more effective use of this insecticide in management programs for *M*. *persicae* through improved understanding of its activity profiles.

## Materials and methods

### Insects

The colony of *M*. *persicae* was established from apterous adults collected from Chinese cabbage (*Brassica oleracea* var. *capitata* L.) field in Fujian Province, China (Site: 26.90° N, 119.46° E) in February 2017. The insects were reared on vermiculite-cultured radish (*Raphanus sativus* L.) seedlings and maintained at 23 ± 1°C, 65–75% relative humidity (RH), with a photoperiod of 16: 8 (L: D) h in the laboratory.

### Insecticide and solutions

Flupyradifurone (CAS number: 951659-40-8) at the 96% of active ingredient was obtained from Bayer CropScience Co. Ltd (Monheim, Germany). Triton X-100 was purchased from Sigma-Aldrich Co. Ltd (Saint Louis, USA). All other chemicals and solvents used were technique grade reagents. A stock solution of flupyradifurone was prepared in acetone and diluted to appropriate concentrations with 0.05% (v/v) aqueous Triton X-100. In our bioassays, the acetone was controlled less than 1% in all final used insecticide solutions. The control was performed with distilled water containing 0.05% (v/v) Triton X-100.

### Toxicity of flupyradifurone against *M*. *persicae*

A leaf-dip bioassay procedure initially developed by Moores et al. [33] and slightly modified by Tang et al. [17] was used to evaluate the acute toxicity of flupyradifurone to *M*. *persicae*. Briefly, wells of 12-well tissue-culture plates purchased from Corning (NY, USA) were filled with 2 mL of 2% (w/v) agar to keep the leaves turgid. Leaf discs (20 mm in diameter) were excised from fresh Chinese cabbage using a stainless steel cork borer. Leaf discs were individually dipped to insecticide-free (control) or serial insecticide solutions for 15 s and allowed to air dry for 1 h on disposable plastic gloves (Haimen Yangzi Medical Equipment Co., Ltd, Jiangsu Province, China). After drying, discs were placed upside down on agar in a 12-well tissue-culture plate. Each concentration was conducted three replicates, and 20 apterous adult aphids (≤ 24 h old) were transferred to each well with a soft paintbrush; each bioassay consisted of six concentrations. Each well with aphids was covered with Chinese art paper, also called Xuan paper, purchased from China Xuan Paper Co., Ltd (Anhui Province, China) to prevent aphid escaping. Then the plates with aphids were maintained at the laboratory conditions as described above. Mortality was examined after 48 h. Aphids that unable to move when carefully touched with a soft brush were considered dead. The LC_25_ for subsequent experiments and LC_50_ were calculated using PoloPlus 2.0 software (LeOra Software Inc., Berkeley, CA). The concentration-mortality relationship was considered valid (i.e., they fitted the observed data) when there was absence of significant deviation between the observed and the expected mortality (P > 0.05) [34].

### Sublethal and transgenerational effects of leaf-dip exposure to flupyradifurone

A concentration (LC_25_) of 2 mg/L approximate to 25% mortality level determined in preliminary bioassays was used in leaf-dip exposure experiments to evaluate the sublethal and transgenerational effects of flupyradifurone in *M*. *persicae*.

Chinese cabbage leaf discs (30 mm in diameter) were dipped in LC_25_ of flupyradifurone or control solution for 15 s, air-dried for 1 h, and then placed in a plastic Petri dish (35 mm diameter) contained 2 mL of 2% (w/v) agar. Single apterous adult (≤ 24 h old) was randomly introduced to each treated leaf disc and more than 150 adults were treated in each group. Then, the Petri dishes were held in the laboratory conditions as described above. Mortality of adults was calculated after 48 h. The survivors were transferred to untreated leaf-discs individually for further study of sublethal effects of flupyradifurone in *M*. *persicae*.

In the experiments, the longevity and fecundity of F_0_ adult aphids were recorded daily and newly laid nymphs were removed until the adult aphid died. In the succeeding progeny generations (F_1_ and F_2_), neonate nymphs with less than one-day of age (105 and 107 of F_1_ and 64 and 110 of F_2_ for the control and the flupyradifurone treatment, respectively) were randomly selected from the former generation and placed individually on the untreated Chinese cabbage leaf discs of Petri dishes for evaluating the transgenerational effects of flupyradifurone. Nymphs were observed throughout their development and total duration until adult emergence and survival were record. After the final ecdysis, adult survival and the number of F_1_ or F_2_ progeny were recorded daily until death. Leaf discs were replaced in each dish every five days with freshly untreated leaf discs during the experiments. All experiments were conducted at 23 ± 1°C, 65–75% RH, and a photoperiod of 16: 8 (L: D) h. Age-stage, two-sex life tables were constructed from the data obtained in these experiments.

### Statistical analysis

The raw data for each *M*. *persicae* individual collected in the life table study were analyzed using the age-stage two-sex life table theory [35,36]. The population parameters, including the intrinsic rate of increase (*r*), finite rate of increase (*λ*), net reproductive rate (*R*_0_), the mean generation time (*T*), age-stage specific survival rates (*s*_*xj*_, where *x* is age and *j* is stage), age-specific survival rate (*l*_*x*_), age-specific fecundity (*m*_*x*_), adult pre-reproductive period (APRP), total pre-reproductive period (TPRP), reproductive days (*R*_*d*_) (i.e., the number of days that adult produced offspring), age-specific maternity (*l*_*x*_*m*_*x*_), age-stage specific life expectancy (*e*_*xj*_), reproductive value (*v*_*xj*_), were calculated using the computer program TWOSEX-MSChart [35–37]. The variances and standard errors of the population parameters were estimated using the bootstrap procedure [38] with 100,000 random resampling and difference of population parameters between control and insecticide treatment groups and between generations within each treatment group were compared by using the paired bootstrap test based on the confidence intervals of differences implemented in TWOSEX-MSChart [37,39,40]. All graphics were created using SigmaPlot 12.0 (Systat Software Inc., San Jose, CA, USA).

## Results

### Toxicity of flupyradifurone on *M*. *persicae* adults

The toxicity of flupyradifurone to adult *M*. *persicae* was investigated at 48 h after leaf-dip exposure (Table 1). The LC_50_ value of flupyradifurone was estimated as 8.49 mg L^-1^ with a confidence interval of 5.33–12.39 mg L^-1^ and the LC_25_ value was estimated as 2.10 mg L^-1^ with a confidence interval of 0.99–3.55 mg L^-1^, respectively. The LC_25_ value of 2 mg L^-1^ flupyradifurone was used as the sublethal concentration for the subsequent experiments.

**Table 1:** Toxicity of flupyradifurone to adult *Myzus persicae* determined by using leaf-dip bioassay.

Insecticide	N	Slope ± SE^a^	LC_25_ (95%CI)^b^ mg L^-1^	LC_50_ (95%CI)^b^ mg L^-1^	χ^2^ (*df*)^c^	*P*
Flupyradifurone	665	1.11 ± 0.12	2.10 (0.99–3.55)	8.49 (5.33–12.39)	17.97 (27)	0.91

^a^ Standard error.

^b^ 95% confidence intervals.

^c^ Chi-square value (*χ*^2^) and degrees of freedom (*df*) as calculated by PoloPlus 2.0.

### Sublethal effects of flupyradifurone on the longevity and fecundity of parental (F_0_) *M*. *persicae*

**Fig 1:**
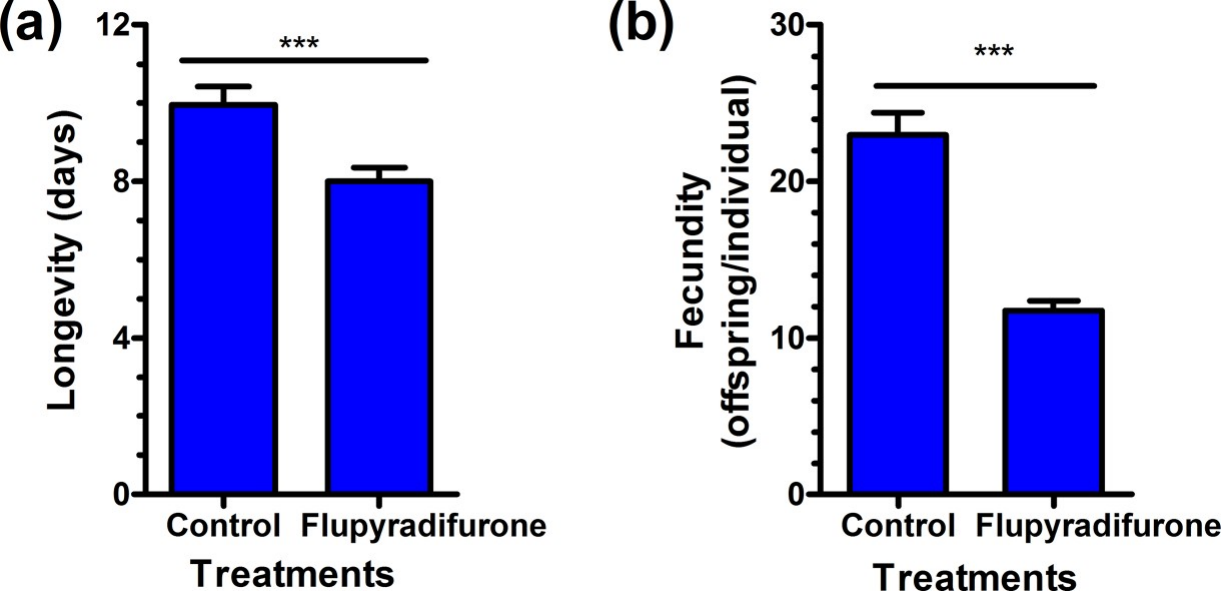
The longevity and fecundity of initial (F_0_) adult *Myzus persicae* treated with LC_25_ of flupyradifurone for 48 h. The *** above bars indicate means are significant difference between the treatment and the control based on paired bootstrap test (*P* < 0.001).

Short-term exposure (48 h) of adult *M*. *persicae* to LC_25_ of flupyradifurone on leaf discs had a significant effect on the longevity and fecundity of the exposed individuals (F_0_ generation) (Fig 1). As compared to the control group, the adult longevity of the F_0_ was significantly reduced from 9.96 d to 8.01 d by flupyradifurone treatment (*P* < 0.001; Fig 1). The fecundity of F_0_ adults were also significantly reduced from 22.96 to 11.75 offspring/female after exposure to LC_25_ of flupyradifurone (*P* < 0.001; Fig 1).

### Transgenerational effects of flupyradifurone on development, longevity and fecundity of *M*. *persicae*

The development time, longevity, fecundity and total preadult survival rate of the succeeding progeny generations (F_1_ and F_2_) were evaluated (Table 2). The sublethal concentration of flupyradifurone had significant effects on the development time and fecundity of F_1_ generation *M*. *persicae* (Table 2). The duration of first instar nymph (*P* = 0.0075) and reproductive days (*P* = 0.0006) of F_1_ individuals were significantly prolonged by flupyradifurone treatment, as well as the fecundity of F_1_ individuals was significantly stimulated by the sublethal flupyradifurone from 26.60 ± 1.32 to 35.25 ± 2.19 offspring/female (*P* = 0.0006; Table 2). Whereas, when compared to those of the control, the 48-h exposure of F_0_ adult *M*. *persicae* to flupyradifurone significantly decreased the duration of third instar and fourth instar nymph, preadult duration, adult pre-reproductive period (APRP) and total pre-reproductive period (TPRP) in F_1_ individuals (*P* < 0.01; Table 2) and significantly decreased the adult longevity, reproductive days and fecundity in F_2_ individuals (*P* < 0.01; Table 2). However, no significant differences of F_1_ generations were observed for the duration time of the second instar nymph, adult longevity, total longevity or total preadult survival rate between the control and flupyradifurone treatments. The total longevity, total preadult survival rate and the duration time of each instar nymph stage, preadult, APRP and TPRP of F_2_ individuals in flupyradifurone treatment were not significantly different from the control (*P* > 0.05; Table 2).

**Table 2:** Transgenerational effects on developmental time, longevity, adult pre-reproductive period (APRP), total pre-reproductive period (TPRP), total preadult survival rate, and mean fecundity of the succeeding generations after initial adult *Myzus persicae* 48-h exposed to LC_25_ of flupyradifurone.

Biological parameters	Generation	Control		Flupyradifurone LC_25_	
		N	Mean ± SE^a^^,^ ^b^	N	Mean ± SE^a^^,^ ^b^
First instar (d)	F_1_	94	1.99 ± 0.06bA	91	2.23 ± 0.07aA
	F_2_	60	1.52 ± 0.07aB	107	1.67 ± 0.06aB
Second instar (d)	F_1_	91	1.75 ± 0.07aA	87	1.75 ± 0.08aA
	F_2_	58	1.48 ± 0.07aB	104	1.56 ± 0.06aA
Third instar (d)	F_1_	89	1.99 ± 0.09aA	86	1.60 ± 0.07bA
	F_2_	58	1.48 ± 0.07aB	102	1.47 ± 0.05aA
Fourth instar (d)	F_1_	87	2.24 ± 0.09aA	85	1.56 ± 0.07bA
	F_2_	56	1.77 ± 0.07aB	99	1.68 ± 0.05aA
Pre-adult (d)	F_1_	87	7.87 ± 0.19aA	85	7.11 ± 0.10bA
	F_2_	56	6.29 ± 0.11aB	99	6.34 ± 0.07aB
Adult longevity (d)	F_1_	87	12.53 ± 0.43aB	85	13.75 ± 0.67aA
	F_2_	56	14.69 ± 0.78aA	99	12.07 ± 0.45bB
Total longevity (d)	F_1_	105	17.56 ± 0.72aA	107	17.19 ± 0.88aA
	F_2_	64	18.72 ± 1.02aA	110	17.00 ± 0.57aA
APRP (d)	F_1_	85	1.27 ± 0.09aA	79	0.87 ± 0.07bB
	F_2_	55	1.02 ± 0.09aB	99	1.08 ± 0.07aA
TPRP (d)	F_1_	85	9.14 ± 0.24aA	79	7.94 ± 0.11bA
	F_2_	55	7.29 ± 0.10aB	99	7.42 ± 0.09aB
Reproductive days (d)	F_1_	87	10.02 ± 0.29bB	85	12.08 ± 0.51aA
	F_2_	56	12.29 ± 0.66aA	99	9.98 ± 0.39bB
Total preadult survival	F_1_	105	0.83 ± 0.04aA	107	0.79 ± 0.04aB
	F_2_	64	0.87 ± 0.04aA	110	0.90 ± 0.03aA
Fecundity (offspring/female)	F_1_	87	26.60 ± 1.32bB	85	35.25 ± 2.19aA
	F_2_	56	39.52 ± 2.73aA	99	32.77 ± 1.63bA

^a^ Standard errors (SE) were estimated by using the bootstrap technique with 100,000 re-samplings.

^b^ Significant difference at *P* < 0.05 between two different treatments and generations were compared with paired bootstrap test implemented in TWOSEX-MSChart. The lower-case letters show significant differences between control and flupyradifurone treatments in the same generation, while the capital letters indicate the significant differences between generations within the same treatment (*P* < 0.05).

In addition, the duration of first instar nymph, pre-adult period, adult longevity, TPRP, reproductive days and fecundity of F_2_ individuals were significantly lower than that of F_1_ individuals in the flupyradifurone treatment (*P* < 0.05; Table 2); By contrast, the APRP and total preadult survival rate of F_2_ individuals were significant higher than that of F_1_ individuals and no significant difference in duration time of second instar and third instar nymph and total longevity were observed between F_1_ and F_2_ generations in the flupyradifurone treatment group (*P* < 0.05; Table 2). Similarly, the duration time of each juvenile developmental stage (including 1^st^, 2^nd^, 3^rd^ and 4^th^ instar nymph stage and preadult period) and pre-reproductive period (including APRP and TPRP) of F_2_ individuals were significantly lower than that of F_1_ individuals in the control group (*P* < 0.05; Table 2); Whereas, the adult longevity, reproductive days and fecundity of F_2_ individuals were significantly higher than that of F_1_ individuals in the control (*P* < 0.05; Table 2).

### Transgenerational effects of flupyradifurone on population parameters of *M*. *persicae*

The transgenerational effects of flupyradifurone (LC_25_) on the population parameters of F_1_ and F_2_ generations were evaluated with bootstrap technique based on life tables (Table 3). When compared to the control group, the net reproductive (*R*_0_) of F_1_ generation *M*. *persicae* was significantly increased after the sublethal flupyradifurone treatment (*P* = 0.0245; Table 3), and the intrinsic rate of increase (*r*) and finite rate of increase (*λ*) of F_1_ were not significantly affected by flupyradifurone, although a stimulation tendency was observed in these two parameters (*P* = 0.0628; Table 3).The mean generation time (*T*) was not significantly affected in F_1_ generation (*P* = 0.893; Table 3). However, compared with the control, the *T* of F_2_ individuals was significantly decreased in the flupyradifurone treatment (*P* = 0.0084; Table 3). The *R*_0_ of F_2_ individuals was also showed a decline tendency from 34.56 in the control to 29.50 offspring/female in the flupyradifurone treatment, while the difference was not significant (*P* = 0.1313; Table 3). In addition, the *r* and *λ* of F_2_ generation were not significantly affected by flupyradifurone as compared to that of the control (*P* = 0.9420 for *r*, *P* = 0.9407 for *λ*; Table 3). In addition, except no significant difference was observed in *R*_0_ of the flupyradifurone treatment group, the *r*, *λ* and T were significantly increased from F_1_ to F_2_ generation within the control or the flupyradifurone treatment, as well as *R*_0_ of F_2_ was higher than *R*_0_ of F_1_ in the control (*P* < 0.001; Table 3).

**Table 3:** Population parameters of the succeeding generations after initial adult *Myzus persicae* exposed to LC_25_ of flupyradifurone for 48 h.

Population parameter^a^	Generation	Bootstrap (mean ± SE^b^^,^ ^c^)	
		Control	Flupyradifurone
*r* (d^−1^)	F_1_	0.2392 ± 0.0069aB	0.2570 ± 0.0067aB
	F_2_	0.2956 ± 0.0008aA	0.2949 ± 0.0053aA
*λ* (d^−1)^	F_1_	1.270 ± 0.009aB	1.293 ± 0.009aB
	F_2_	1.344 ± 0.010aA	1.343 ± 0.007aA
*R*_0_ (offspring/female)	F_1_	22.03 ± 1.46bB	28.00 ± 2.22aA
	F_2_	34.55 ± 2.86aA	29.50 ± 1.74aA
*T* (d)	F_1_	12.92 ± 0.18aA	12.96 ± 0.15aA
	F_2_	11.97 ± 0.15aB	11.47 ± 0.12bB

^a^ *r*, intrinsic rate of increase; *λ*, finite rate of increase; *R*_0_, net reproductive rate; *T*, mean generation time.

^b^ Standard errors (SE) were estimated by using the bootstrap technique with 100,000 re-samplings.

^c^ Significant difference at *P* < 0.05 between two different treatments and generations were compared with paired bootstrap test implemented in TWOSEX-MSChart. The small letters show significant differences between control and flupyradifurone treatments in each generation, while the capital letters indicate the significant differences between F_1_ and F_2_ generations within each treatment groups (*P* < 0.05).

### Transgenerational effects of flupyradifurone on age-stage specific survival rate and fecundity of *M*. *persicae*

**Fig 2:**
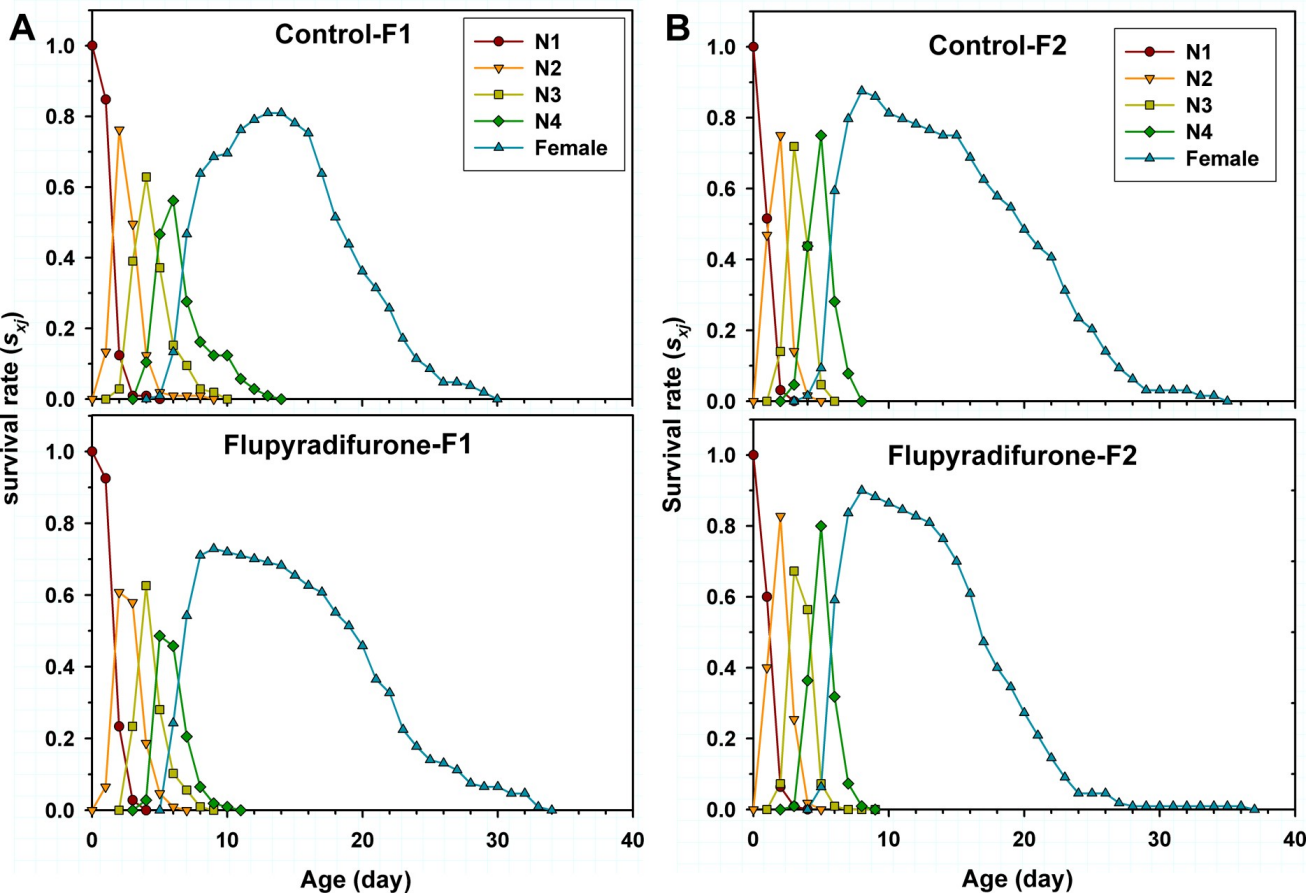
Age-stage-specific survival rates (*s*_*xj*_) of *Myzus persicae* treated with LC_25_ of flupyradifurone in F_1_ and F_2_ generations.

Age-stage survival rate curves (*s*_*xj*_) show the probability that a newborn nymph will survive to age *x* and stage *j*. Obvious overlaps between different stages occurred in both flupyradifurone-treated and the control groups as a result of the variable developmental rates among individuals (Fig 2). Declined survival rates of each developmental stage of F_1_ individuals were observed in the flupyradifurone treatment group as compared to the control group (Fig 2), however, the survival rates of the 2nd- (0.83) and 4th- (0.80) instar nymph of F_2_ individuals in the flupyradifurone treatment were higher than that of the control (0.75 and 0.44 for 2^nd^ and 4^th^ instar nymph, respectively; Fig 2). The fourth instar nymph peak and the female adult peak of F_1_ generation appeared at five days of age and nine days of age in the flupyradifurone treatment, while in the control at six days of age and at thirteen days of age, respectively (Fig 2). These showed the faster development in the flupyradifurone treatment group. Therefore, total development time of F_1_ generational nymph in the flupyradifurone treatment (10 days) was shorter than that of the control (13 days; Fig 2). For F_2_ generation, a similar trend of *s*_*xj*_ curves was observed between the control and flupyradifurone treatment groups (Fig 2). In addition, between generations, the adult survival rate of F_1_ individuals was lower than F_2_ individuals in each treatment group (Fig 2).

**Fig 3:**
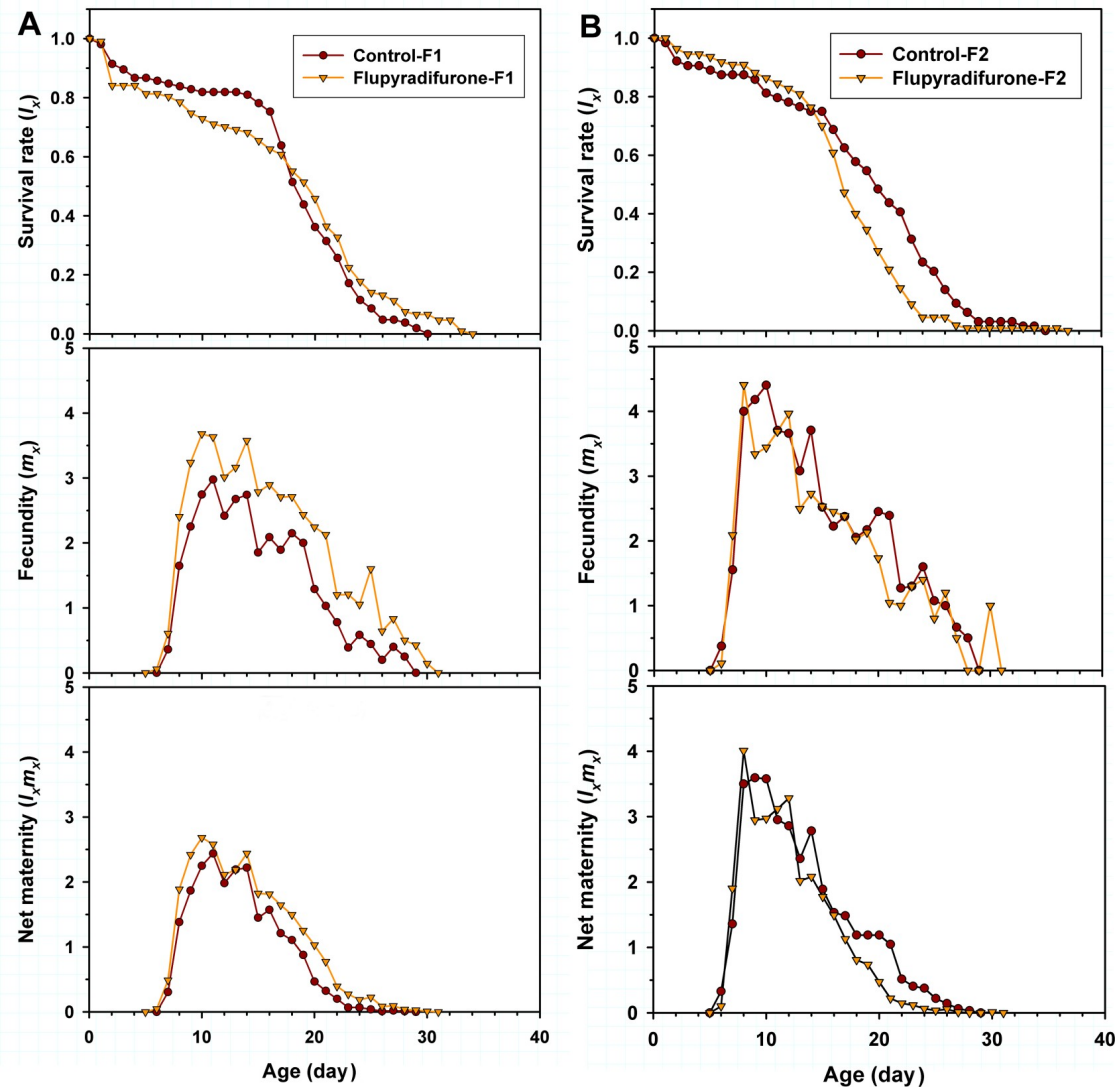
Age-specific survival rate (*l*_*x*_), age-specific fecundity of total population (*m*_*x*_), and age-specific maternity (*l*_*x*_*m*_*x*_) of initial *Myzus persicae* exposed to LC_25_ of flupyradifurone in F_1_ and F_2_ generations.

The age-specific survival rate (*l*_*x*_) demonstrates a simplified overview of the survival rate without accounting for the stage differentiation (Fig 3). The *l*_*x*_ curves significantly declined on day 15 or 14 in F_1_ or F_2_ generation in both control and flupyradifurone treatment groups (Fig 3). Interesting, a higher *l*_*x*_ of F_1_ generation were observed in flupyradifurone treated group from age 18 to 33 day (Fig 3), whereas a lower *l*_*x*_ of F_2_ generation in the flupyradifurone treatment was observed from age 15 to 36 day (Fig 3). The age-specific fecundity of the total population (*m*_*x*_) and age-specific maternity (*l*_*x*_*m*_*x*_) of the flupyradifurone treated *M*. *persicae* were higher than that of the control group in F_1_ generation (Fig 3), while a similar trend of *m*_*x*_ and *l*_*x*_*m*_*x*_ of F_2_ generation *M*. *persicae* were observed both in the treatment and the control groups (Fig 3).

**Fig 4:**
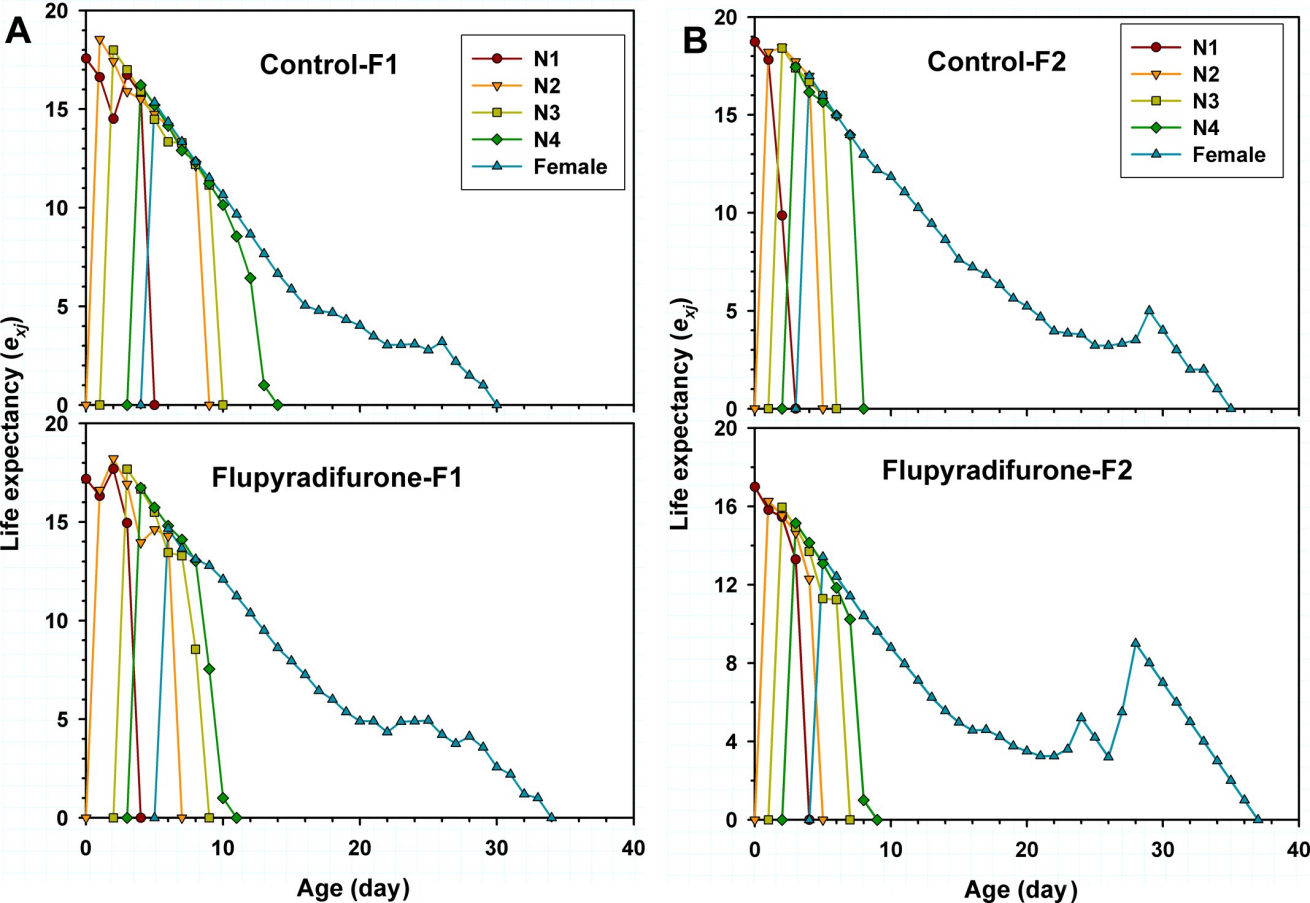
Age-stage-specific life expectancy (*e*_*xj*_) of *Myzus persicae* treated with LC_25_ of flupyradifurone in F_1_ and F_2_ generations.

**Fig 5:**
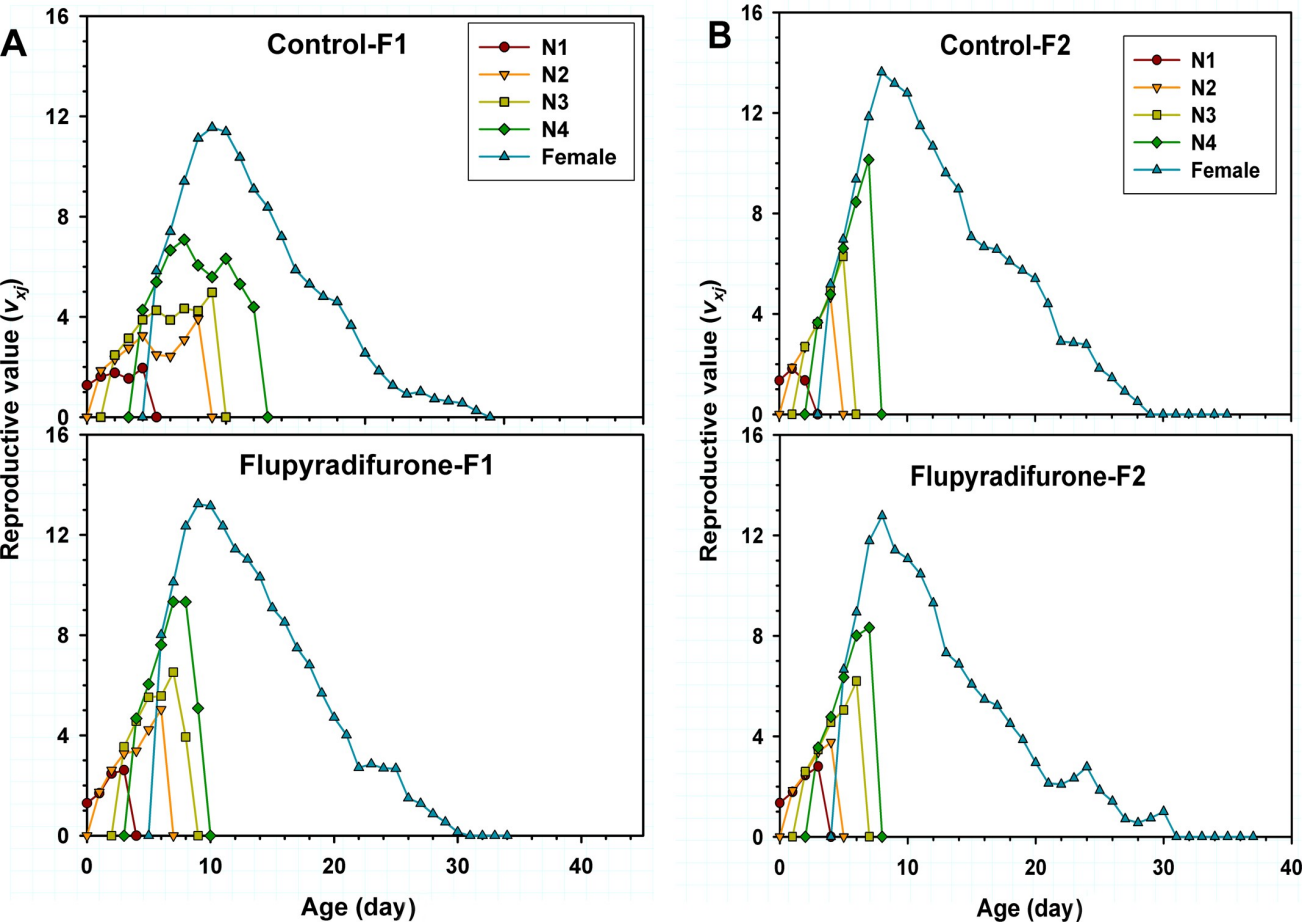
Age-stage reproductive value (*v*_*xj*_) of *Myzus persicae* treated with LC_25_ of flupyradifurone in F_1_ and F_2_ generations.

The age-stage-specific life expectancy (*e*_*xj*_) is the length of time that an individual of age *x* and stage *j* is expected to survive after age *x* (Fig 4). The life expectancy (*e*_*xj*_) curves indicated that offspring (F_1_ and F_2_) of adult *M*. *persicae* with one-time flupyradifurone exposure could to survive longer than the control (Fig 4). In addition, the age-stage-specific reproductive value (*v*_*xj*_) represents the devotion to future offspring of individuals from age *x* to stage *j*. A higher maximum reproductive value of each stage and a shorter preadult period of F_1_ generation *M*. *persicae* were observed in the treatment group than those of the control (Fig 5), while in F_2_ generation, except the 1st instar nymph, a lower *v*_*xj*_ of each stage in the treatment group was observed than that of the control (Fig 5).

## Discussion

The high toxicity of flupyradifurone has been reported for several sucking pests, including *M*. *persicae*, *Aphis gossypii* Glover (Hemiptera: Aphididae) and *Bemisia tabaci* Gennadius (Hemiptera: Aleyrodidae) [7]. In this study, flupyradifurone also showed a highly acute toxicity against adult *M*. *persicae* after 48 h leaf-dip exposure and the LC_50_ was 8.491 mg/L and this LC_50_ value is similar to that previously reported for *Diaphorina citri* Kuwayama (Hemiptera: Psyllidae), after 48 h leaf-dip exposure to flupyradifurone with 10.43 mg/L [41]. In addition to the lethal effects of insecticides, insect populations are frequently exposed to low concentrations of insecticides in the field due to the variable distribution and continuous degradation of insecticides [42,43]. Thus, sublethal effects of insecticides may increase or decrease insect population [10], assessing the development, survival, reproduction and behavioral response are important for overall understanding of the effects of flupyradifurone for IPM. In this study, an attempt was made to assess sublethal effects on life table characteristics of *M*. *persicae* over subsequent generations after exposed *M*. *persicae* adult to sublethal flupyradifurone for 48 h.

Sublethal effects that reduced fecundity and longevity, and altered behavior were usually observed in most insect pests after exposure to sublethal concentration of insecticides [10,44–46]. For example, endosulfan can significantly reduce fecundity of *Apolygus lucorum* Meyer-Dür (Hemiptera: Miridae) after treated with sublethal concentrations [47] and buprofezin reduce adult longevity of *B*. *tabaci* by sublethal doses [48]. Similarly, in the present study, when initial *M*. *persicae* adult was exposed to leaf discs treated with sublethal concentrations of flupyradifurone, significant reductions in the fecundity and adult longevity of F_0_ generation *M*. *persicae* were observed and the reproductive days, adult longevity, and fecundity were significantly shortened or reduced in the subsequent F_2_ generation, although no significant effects on the development time of each nymph stage and survival rates were found in F_2_. Moreover, these adverse effects on F_2_ individual aphids were translated to their population parameters including a lower *R*_0_ and mean generation time (*T*) and showed that sublethal concentrations of flupyradifurone suppressed the population growth of F_2_ generation *M*. *persicae*. Similar sublethal effects of insecticides on population growth have been reported in several insect pests, such as *A*. *gossypii* [49], *A*. *lucorum* [50], *B*. *tabaci* [51], *Brevicoryne brassicae* L. (Hemiptera: Aphididae) [52], *Bradysia odoriphaga* Yang et Zhang (Diptera: Sciaridae) [53] and *M*. *persicae* [54,55]. The negative sublethal effects may be due to increased biological fitness cost and also provide evidence that sublethal concentrations of flupyradifurone did have significantly sublethal and transgenerational effects on *M*. *persicae*.

Insecticide-induced hormesis that increased fecundity or change insect behavior have been reported in *A*. *gossypii* with bifenthrin [56], citrus thrip, *Scirtothrips citri* Moulton (Thysanoptera: Thripidae), with dicofol or malathion [57], mite, *Tetranychus urticae* Koch (Acari: Tetranychidae), with imidacloprid [58], and the brown planthopper, *Nilaparvata lugens* Stäl (Hemiptera: Delphacidae) [59,60] after exposure to low or sublethal concentrations of insecticides. In the present study, significantly increased fecundity and more reproductive days were observed in F_1_ adults, indicating that sublethal concentration of flupyradifuron has a stimulatory effect (i.e., hormesis) on reproduction of F_1_ generation *M*. *persicae* following parental adults 48-h exposure. Similar stimulatory effects on reproduction of *M*. *persicae* in offspring generations have been documented in response to sublethal concentration of several insecticides, including azinphosmethyl [61], azadirachtin [55], imidacloprid [55,62], and sulfoxaflor [17]. Additionally, in the present study, the sublethal concentration of flupyradifuron significantly affected the F_1_ generation population growth, notably through a significantly shortened duration of third instar and fourth instar nymph, preadult period and pre-reproductive periods (APRP and TPRP), a significantly prolonged reproductive days and an increased fecundity. The sum of these effects on F_1_ individual aphids translated to higher population parameters, including net reproductive rate (*R*_0_), intrinsic rate of increase (*r*) and finite rate of increase (λ). These increased population parameters and stimulated reproduction indicated that sublethal concentration of flupyradifuron could to stimulate population growth of *M*. *persicae* in F_1_ generation via maternal effects. Similarly, the reproduction of *Daphnia carinata* King (Diplostraca: Daphnidae) was negatively affected in the parental generation following exposure to low concentrations of chlorpyrifos, while a hormesis effect was observed for all reproductive parameters in the second generation [63] and the females of *Chironomus riparius* Kieffer (Diptera: Chironomidae) exposed to low tributylin concentrations for multiple generations significantly laid more eggs in the subsequent generations and acquired a higher tolerance towards the stressor [64]. These results suggested that sublethal effects on biological fitness may increase by exposure to low concentrations of insecticides.

Interestingly, in the present study, 48-h exposures of adult *M*. *persicae* (F_0_) to sublethal concentration of flupyradifuron can significantly stimulate fecundity in the following F_1_ generation, whereas reduced fecundity and adult longevity were observed in F_0_ and F_2_ generations, and no significant effects on development time of each instar nymph and population growth (*R*_0_) were found between the treatment and control in F_2_ generations. These results suggested that biological tradeoffs in resource allocation occurred across generations. Similarly, when *M*. *persicae* adults exposed to low/sublethal concentrations of azinphos-methyl [65], imidacloprid [15,62] and sulfoxaflor [17], this delayed stimulation of reproduction phenomenon was also observed in the subsequent generation of *M*. *persicae*, while not observed in initial adults. We hypothesized that the exposure of adult *M*. *persicae* to sublethal concentration of insecticides may: remove individuals of low fitness, cost biological fitness to cope with the stress of flupyradifurone in F_0_; and result in stimulating reproduction (i.e. hormesis) and higher reproduction of F_1_ progeny to overcompensate physiologically for their disrupted homeostasis via maternal effects, to optimize resource allocation between self-maintenance and reproductive output; and possibly restore homeostasis in F_2_ generation. This no long-term fitness cost for the stimulatory response in early generations was also been demonstrated for imidacloprid in *M*. *persicae* [23,66]. Therefore, hormesis induced by sublethal concentrations of flupyradifuron may lead to secondary population outbreaks of *M*. *persicae*.

In conclusion, our results indicated that LC_25_ concentration of flupyradifurone has transgenerational hormesis on *M*. *persicae* across three generations. The sublethal exposure of parental aphids resulted in significantly increase of duration of 1st instar nymph, reproductive days, fecundity and population parameters *R*_0_ in F_1_ generation and recovery *R*_0_ to control level and reduced *T* in F_2_ generation. These suggested that short-term exposure of the sublethal concentration of flupyradifurone might induce hormesis of *M*. *persicae* and this hormesis may lead to pest resurgence [57,67]. Because hormetic effects can be taken in form of shortened development, higher survival rate, or higher fecundity, these factors are not independent from each other and should not be analyzed separately [68,69]. Since life table analysis integrated all these factors in population parameters, it is the most important tool for an overall evaluation of population fitness and hormesis [17,70]. Nevertheless, given that the genetic variation in field populations is naturally greater than that of laboratory strains, the situation in the field may be more complex and further investigations on sublethal effects of this insecticide on *M*. *persicae* in the field are advisable. Consequently, flupyradifurone was very toxic against *M*. *persicae M*. *persicae* at the present study, but insecticide-induced hormesis should be taken into consideration, which may potentially occur after application of flupyradifurone for control *M*. *persicae* in the field.

## Supporting information

S1 File Lifetable of F_0_-Control. (XLSX)

S2 File Lifetable of F_0_-Flupyradifurone. (XLSX)

S3 File Lifetable of F_1_ progeny produced by F_0_-Control adults (F_1_-Control). (XLSX)

S4 File Lifetable of F_1_ progeny produced by F_0_-flupyradifurone adults (F_1_-flupyradifurone). (XLSX)

S5 File Lifetable of F_2_ progeny produced by F_1_-Control adults (F_2_-Control). (XLSX)

S6 File Lifetable of F_2_ progeny produced by F_1_-flupyradifurone adults (F_2_-Flupyradifurone). (XLSX)
